# Linking Microbial Community Structure and Function During the Acidified Anaerobic Digestion of Grass

**DOI:** 10.3389/fmicb.2018.00540

**Published:** 2018-03-21

**Authors:** Aoife Joyce, Umer Z. Ijaz, Corine Nzeteu, Aoife Vaughan, Sally L. Shirran, Catherine H. Botting, Christopher Quince, Vincent O’Flaherty, Florence Abram

**Affiliations:** ^1^Functional Environmental Microbiology, School of Natural Sciences, National University of Ireland Galway, Galway, Ireland; ^2^Environmental Omics Laboratory, School of Engineering, University of Glasgow, Glasgow, United Kingdom; ^3^Microbial Ecology Laboratory, School of Natural Sciences, National University of Ireland Galway, Galway, Ireland; ^4^Biomedical Sciences Research Complex, University of St Andrews, Fife, United Kingdom; ^5^Microbiology and Infection, Warwick Medical School, University of Warwick, Coventry, United Kingdom

**Keywords:** anaerobic digestion, cellulosic substrate, 16S rRNA profiling, metaproteomics, biomolecule co-extraction

## Abstract

Harvesting valuable bioproducts from various renewable feedstocks is necessary for the critical development of a sustainable bioeconomy. Anaerobic digestion is a well-established technology for the conversion of wastewater and solid feedstocks to energy with the additional potential for production of process intermediates of high market values (e.g., carboxylates). In recent years, first-generation biofuels typically derived from food crops have been widely utilized as a renewable source of energy. The environmental and socioeconomic limitations of such strategy, however, have led to the development of second-generation biofuels utilizing, amongst other feedstocks, lignocellulosic biomass. In this context, the anaerobic digestion of perennial grass holds great promise for the conversion of sustainable renewable feedstock to energy and other process intermediates. The advancement of this technology however, and its implementation for industrial applications, relies on a greater understanding of the microbiome underpinning the process. To this end, microbial communities recovered from replicated anaerobic bioreactors digesting grass were analyzed. The bioreactors leachates were not buffered and acidic pH (between 5.5 and 6.3) prevailed at the time of sampling as a result of microbial activities. Community composition and transcriptionally active taxa were examined using 16S rRNA sequencing and microbial functions were investigated using metaproteomics. Bioreactor fraction, i.e., grass or leachate, was found to be the main discriminator of community analysis across the three molecular level of investigation (DNA, RNA, and proteins). Six taxa, namely Bacteroidia, Betaproteobacteria, Clostridia, Gammaproteobacteria, Methanomicrobia, and Negativicutes accounted for the large majority of the three datasets. The initial stages of grass hydrolysis were carried out by Bacteroidia, Gammaproteobacteria, and Negativicutes in the grass biofilms, in addition to Clostridia in the bioreactor leachates. Numerous glycolytic enzymes and carbohydrate transporters were detected throughout the bioreactors in addition to proteins involved in butanol and lactate production. Finally, evidence of the prevalence of stressful conditions within the bioreactors and particularly impacting Clostridia was observed in the metaproteomes. Taken together, this study highlights the functional importance of Clostridia during the anaerobic digestion of grass and thus research avenues allowing members of this taxon to thrive should be explored.

## Introduction

The development of the bioeconomy is critical in attaining several of the UN Sustainable Development Goals for 2030 (United Nations, 2015), including SDG7 (renewable energy), SDG8 (good jobs and economic growth) and SDG13 (climate action), as well as achieving 20% energy production in Europe from renewable sources by 2020 (Vega et al., 2014). In this context harvesting valuable bioproducts from various waste streams becomes a necessary element for the growth of a sustainable bioeconomy worldwide (Werner et al., 2011). Anaerobic digestion (AD) is a well-established sustainable technology for the treatment of a diverse range of wastewaters (Adulkar and Rathod, 2014; Mustafa et al., 2014; Yuan et al., 2014; Lackey et al., 2015; Wang et al., 2015; Zerrouki et al., 2015), as well as solid wastes (Wang et al., 2014; Michele et al., 2015; Nielfa et al., 2015; Ratanatamokul and Saleart, 2016) converting waste streams to energy produced in the form of biogas.

Recently, there has been a considerable increase in energy crops usage in the biogas industry (Vega et al., 2014) with corn representing one of the favorite feedstocks. However, using such crops for energy production is directly competing with other industries (Ranum et al., 2014). In addition, the crops require close management, as they have to be replanted and typically involve the use of fertilizers and pesticides for successful growth. Therefore alternative feedstocks need to be identified as bioproducts sources. Grass is such an alternative feedstock, as it grows naturally in many areas, with minimal labor necessary to sustain it. Grass is estimated to represent 70% of agricultural land worldwide and cover 40% of terrestrial surfaces (Cerrone et al., 2014). It can grow on soils unsuitable for other crops and its production has been estimated, in Ireland for example, to exceed livestock requirements by about 1.7 million tons dry solids each year (McEniry et al., 2013). As such, grass represents a promising second-generation biomass resource. Perennial ryegrass has been demonstrated to be a suitable feedstock for AD (Cysneiros et al., 2011), leading to the production of energy with the potential for recovery of process intermediates of high market values (Cerrone et al., 2014).

The natural process of AD is driven by the concerted, sequential and cooperative activities of several microbial trophic groups. Broadly, four main steps can be distinguished during the process: (i) hydrolysis where polymers are converted to monomers; (ii) acidogenesis leading to volatile fatty acid production; (iii) acetogenesis leading to acetate and H_2_/CO_2_ generation and finally; (iv) methanogenesis where acetate and H_2_/CO_2_ are converted to CH_4_ (Narihiro and Sekiguchi, 2007). Even though microbial consortia clearly underpin AD, the relationship between process performance and microbial community composition and functioning have yet to be adequately characterized (Amha et al., 2018). Reactors are typically designed and operated on the basis of empirical relationships between reactor performance and process parameters, bypassing microbial processes undeniably at the core of AD. As a result, process instability and failures, due to, for example, the accumulation of free ammonia, volatile fatty acids, long chain fatty acids and low pH, are still common and poorly understood (Amha et al., 2018). Thus the advancement of the technology relies on a greater insight and understanding of the behavior of the AD microbiome. There is, however, limited knowledge of the functional activities of the microbial consortia present in AD systems (Abram et al., 2011; Abdul et al., 2014), and this is especially true for the AD of solid feedstocks.

Recent technological developments, and specifically the advancement of high-throughput omics, have allowed for the possibility of system approaches to be explored (Siggins et al., 2012a; Abram, 2015; Narayanasamy et al., 2015). Particularly, metaproteomics can be used to determine key metabolic pathways and functional activities occurring in a given ecosystem at the time of sampling. Proteins identified can support the characterization of microbial groups involved in specific functions via protein assignment. Metaproteomics has been applied to many diverse environments including marine, freshwater, soil, human biology as well as natural and bioengineered systems (Siggins et al., 2012b; Wilmes et al., 2015). It has also been previously employed to uncover key biochemical metabolic pathways occurring in anaerobic bioreactor treating wastewater (Abram et al., 2011; Siggins et al., 2012b; Hettich et al., 2013; Gunnigle et al., 2015a,b; Heyer et al., 2016) and more recently solid feedstocks (Kohrs et al., 2014; Lü et al., 2014; Theuerl et al., 2015; Heyer et al., 2016; Abendroth et al., 2017). Here, we report on the investigation of the microbial community structure and function in triplicate anaerobic bioreactors digesting grass. The leachates of the reactors were not buffered, in order to favor the accumulation of process intermediates as a result of methanogenesis inhibition via acidification. 16S rRNA amplicon profiling from DNA and cDNA samples was combined with metaproteomics in an effort to link the knowledge obtained from sequencing data (community structure) to the functional activities taking place at the time of sampling.

## Materials and Methods

### Bioreactor Operation and Sampling

Triplicate leach-bed bioreactors (R1, R2, and R3), with a working volume of 4 L, were operated at 37°C in a semi-continuous mode with a solid retention time of 7 days as previously described (Cysneiros et al., 2012). For the first batch, the triplicate bioreactors were seeded with 84 g volatile solids (VS) of pressed ensiled ryegrass and 126 g VS of anaerobic granular sludge from a full-scale mesophilic reactor (Carbery Milk Products, Ireland) to which 3.2 L of water supplemented with trace elements (0.2 mM MnCl_2_, 0.2 mM H_3_BO_3_, 0.1 mM ZnCl_2_, 0.06 mM CuCl_2_, 0.01 mM NaHSO_4_, 0.6 mM CaCl_2_, 0.07 mM NiCl_2_ and 0.1 mM SeO_2_) were added. The leachate was recirculated in a down-flow mode using a peristaltic pump. At the end of each bioreactor run (7 days), 126 g VS of digestate were used to inoculate the next batch to which 84 g VS of ensiled pressed grass and 1.6 L of leachate from the previous batch supplemented with 1.6 L of freshly prepared leachate (water and trace elements) were added. VS analysis was performed gravimetrically according to the standard method of American Public Health Association [APHA] (2005). At the time of sampling, the bioreactors had been operated for 63 consecutive batches (each of 7 days duration). Duplicate 250 ml leachate and 50 g digestate samples were taken from each of the triplicate bioreactors (R1, R2, and R3) on the last day of the 63rd batch (day 7), when VS removal was 80, 81, and 73% for R1, R2, and R3, respectively. The pH of the reactors was allowed to fluctuate naturally in order to inhibit methane production and in turn favor process intermediates accumulation. A summary of the bioreactors’ performance is presented in **Supplementary Table S1**. Chemical oxygen demand (COD) measurements were performed according to the Standing Committee of Analysts (1985). The leachate samples were centrifuged at 8,000 × *g* for 15 min at 4°C, prior to resuspension in 1 ml of 10 mM Tris Base, 0.1 mM EDTA and 5 mM MgCl_2_ (resuspension buffer) and storage on ice before further use. Digestate samples were carefully drained, then immersed in 250 ml of resuspension buffer and placed in a sonication bath for 5 min to gently detach the grass biofilms. After grass removal, the digestate samples were filtered through two layers of muslin cloth twice before centrifugation at 8,000 × *g* for 15 min at 4°C. The resulting pellets were resuspended in 1 ml of resuspension buffer. Leachate and digestate samples were then centrifuged at 17,000 × *g* for 10 min before undergoing the following series of washes (Roume et al., 2012): twice with 1 ml of 0.9% NaCl, then twice with 1 ml 50 mM Tris-HCl and finally once with 1 ml resuspension buffer. The pellets were then flash frozen in liquid nitrogen and stored at -80°C until further use.

### High-Throughput 16S rRNA Sequencing and Bioinformatic Analysis

DNA, RNA, and proteins were co-extracted from digestate and leachate samples using the RNA/DNA/Protein Purification kit from Norgen Biotek. Briefly, digestate and leachate cell pellets were resuspended in 500 μl lysis solution and 500 μl 1X Tris-EDTA to which 10 μl ml^-1^ β-mercaptoethanol were added. Cell lysis was carried out by bead beating for 30 s using zirconia beads (0.5 ml: 0.1 mm and 0.5 mm diameter in 1:1 ratio). The samples were then centrifuged at 17,000 × *g* for 30 min, and this step was repeated until no pellet was visible. The resulting supernatants were supplemented with 100 μl pure ethanol and loaded onto all-in-one chromatographic spin columns (Norgen Biotek). Purification and isolation of DNA, RNA and proteins were carried out following the manufacturer’s recommendations. DNase treatment of RNA samples was performed using the Turbo DNA-free kit (Ambion). Control PCRs using DNase treated products as templates were carried out to ensure that no DNA remained in the RNA samples prior to cDNA generation using SuperScript III Reverse Transcriptase (Invitrogen), flash freezing in liquid nitrogen and storage at -80°C. Both cDNA and DNA samples were prepared for paired-end 16S rRNA sequencing using Illumina Miseq platform and Golay barcodes. Amplification of the 16S rRNA gene from DNA and cDNA samples was carried out in triplicate 25 μl reactions using 515F/806R primers (targeting the V4 region; Caporaso et al., 2011) and the Q5^®^ High Fidelity DNA Polymerase kit (New England Biolabs) as follows: 1X Q5^®^ reaction buffer, 200 μM dNTPs, 0.5 μM of each primer, 0.02 U μl^-1^ of Q5^®^ TAQ polymerase and 500 ng of template. PCR conditions consisted of a hotstart at 98°C for 30 s, followed by 30 cycles of denaturation at 98°C for 10 s, annealing at 52°C for 30 s and elongation at 72°C for 30 s and a final elongation step at 72°C for 2 min. The replicate amplicons were then pooled and quantified using the Qubit dsDNA HS Assay kit (Life Technologies) following the manufacturers’ instructions. Samples were normalized to 3 ng μl^-1^ and pooled together, prior to Illumina sequencing. A total of 24 samples were analyzed, corresponding to duplicate samples from both grass biofilms and leachate fractions from the triplicate bioreactors. Illumina sequencing was carried out by the Centre for Genomic Research (Liverpool, United Kingdom) and generated a total of 2.13 10^7^ reads corresponding to 1.14 × 10^7^ and 9.98 × 10^6^ DNA and cDNA sequences, respectively. Only 20 reads were obtained for one of the duplicate grass biofilm cDNA samples from R1, which was therefore dropped from further analysis. Sequencing data were analyzed using the Illumina Amplicon Processing Workflow available at: http://userweb.eng.gla.ac.uk/umer.ijaz/bioinformatics/Illumina_workflow.html. Briefly, paired-end reads were trimmed, overlapped and assembled, prior to OTU clustering and chimera removal using the gold database from UCHIME. Phylogenetic trees and OTU assignments were carried out using MUSCLE. The DNA and cDNA sequences were deposited on NCBI’s Sequence Read Archive under the accession number SRP119456.

### Metaproteomics

Protein concentrations were determined using the Calbiochem Non-Interfering Protein Assay^TM^ kit (Merck KGaA, Darmstadt, Germany), following the manufacturer’s instructions. Protein samples were normalized to a concentration of 1.3 μg μl^-1^ and analyzed by GeLC MS/MS (Dzieciatkowska et al., 2014) as follows: 52 μg of each sample were loaded onto SDS-PAGE and the proteins separated along the length of the gels. The protein samples were fractionated to reduce complexity by excising the top, middle and bottom third of each lane, which were analyzed separately. In-gel digestion, protein reduction and alkylation as well as tryptic digestion were performed prior to peptide extraction with 10% formic acid as previously described (Shevchenko et al., 1996). The resulting peptides were then concentrated using a SpeedVac concentrator (Thermo Savant) before separation on an Acclaim PepMap C18 trap and an Acclaim PepMap RSLC C18 column (Thermo Fisher Scientific), using a nanoLC Ultra 2D plus loading pump and nanoLC AS-2 autosampler (Eksigent, Redwood City, CA, United States). The peptides were then eluted with a gradient of acetonitrile, containing 0.1% formic acid (1–40% acetonitrile in 60 min, 40–99% in a further 10 min, followed by washing with 99% acetonitrile for 5 min before re-equilibration with 1% acetonitrile). The eluate was sprayed into a TripleTOF 5600 electrospray tandem mass spectrometer (Sciex, Foster City, CA, United States) and analysis was carried out in Information Dependent Acquisition (IDA) mode, performing 250 ms of MS followed by 100 ms MS/MS analyses on the 20 most intense peaks. MS/MS data were processed with ProteinPilot v4.5 software (Sciex) using the Paragon search algorithm. The resulting mass spectra were searched against the TrEMBL database, using the following search parameters: cysteine alkylation with iodoacetamide, ‘Gel-based ID’ for ‘Special Factors,’ ‘Biological modifications’ for ‘ID focus,’ and a ‘Thorough’ ‘Search effort.’ NCBI and Swiss-Prot databases searches were also carried out and led to similar results (data not shown). Generalist databases were chosen over custom-build databases, composed of representatives of species identified in the DNA and cDNA gene marker sequencing datasets, to avoid transferring the inherent PCR bias typically associated with 16S rRNA profiling to the metaproteomic analysis. In order to mitigate the number of false positive associated with the use of generalist databases a stringent confidence cut-off of 10% was applied. The mass spectrometry metaproteomics data along with the corresponding FDR analysis of each gel chunk were deposited to the ProteomeXchange Consortium *via* the PRIDE partner repository with the dataset identifier PXD007956. A summary of the number of MS/MS acquired, MS/MS assigned to peptides and the number of distinct peptides for each gel chunk is displayed in **Supplementary Table S2**. A threshold of unused Protscore (from ProteinPilot) of 2 (corresponding to protein detection with ≥99% confidence) and a minimum of two peptides were employed for protein identification. When protein assignment was ambiguous, i.e., when a protein was assigned to multiple species, the lowest common ancestor is reported. Analysis of clusters of orthologous groups (COGs) was carried out using MEGAN5^1^ and an overview of metabolic pathways from which proteins were identified was generated using the Metaproteomics Data Analysis Workflow available at http://userweb.eng.gla.ac.uk/umer.ijaz/bioinformatics/Metaproteomics.html. In this pipeline, the enzyme commission (EC) numbers corresponding to the identified proteins are retrieved when available, MinPath (Ye and Doak, 2009) is used to construct parsimonious pathways and iPath2.0 (Yamada et al., 2011) is employed for pathway visualization. Krona plots were constructed using the Krona template (Ondov et al., 2011) and Circos plots using the Circos online tool developed by Krzywinski et al. (2009).

### Statistical Analyses

Non-metric multidimensional scaling (NMDS) based on Bray–Curtis distances was performed using the statistical program R (R Core Team, 2017) to compare microbial community dissimilarities among grass and leachate samples in (i) DNA and cDNA; and (ii) metaproteomics datasets. The group labels are drawn at mean of the ordination values of the samples for that particular group, and the ellipses represent the 95% confidence interval of the standard error of ordination for a given group. To assess the statistical significance of the sample groupings, an analysis of similarity (ANOSIM) was carried out using R. Ratios were calculated, to compare grass and leachate datasets, using (*n*_c_/*n*)/(*N*_c_/*N*), where *n*_c_ is the number of hits to a given category ‘c’ (i.e., taxonomic assignments) in a specific grass dataset (i.e., DNA, cDNA, or proteins), *n* is the total number of hits in all categories in the same grass dataset, *N*_c_ is the number of hits to that category in the corresponding leachate dataset and *N* is the number of hits in all categories in the same leachate dataset. Ratios smaller than 1 indicate an under-representation of a specific category ‘c’ among grass samples compared to leachate samples, with ratios greater than 1 corresponding to an over-representation of a specific category in the grass samples. Statistical over- and under-representation of a given taxonomic assignment between two datasets was determined by pairwise comparisons using two-tailed Fishers’ exact test with confidence intervals at 99% significance (*P*_adj_ < 0.05).

## Results

### Microbiome Composition, and Transcriptionally and Translationally Active Taxa

**FIGURE 1:**
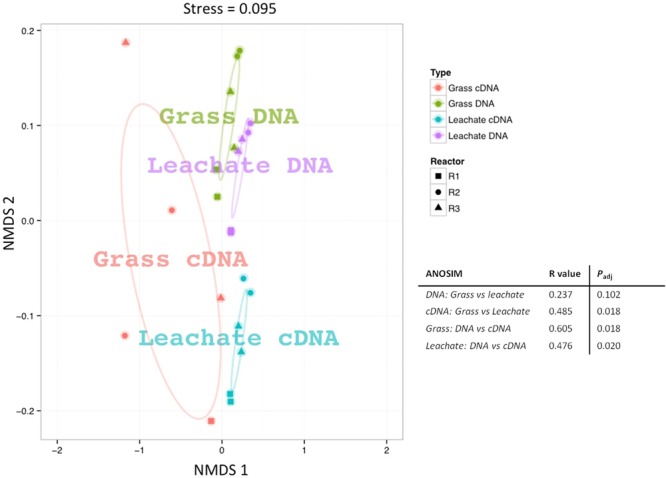
Non-metric multidimensional scaling (NMDS) plot based on Bray–Curtis distances of the 16S rRNA sequences from grass biofilm (DNA represented in green and cDNA in red) and leachate (DNA represented in purple and cDNA in blue) fractions from bioreactors R1 (squares), R2 (circles) and R3 (triangles). Analysis of similarity (ANOSIM) was carried out to assess the statistical significance of sample groupings and the corresponding *R*-values and corrected *P*_adj_-values are displayed.

**FIGURE 2:**
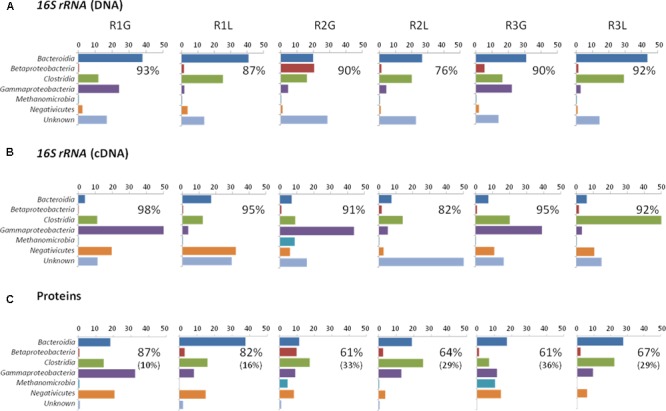
Taxonomic composition, transcriptionally, and translationally active taxa of the grass biofilm and leachate communities from the triplicate reactors R1, R2, and R3. G stands for grass and L for leachate. Community composition analysis was based on taxonomic assignment of **(A)** 16S rRNA gene sequences from DNA samples. Transcriptionally and translationally active taxa analyses were based on taxonomic assignment of **(B)** 16S rRNA gene sequences from cDNA samples and **(C)** proteins. Percentage relative abundances are displayed. The single percentage number displayed in each panel corresponds to the total contribution of the seven taxonomic categories represented (six microbial taxa in addition to unknown) to the datasets. Numbers in brackets represent the percentage of proteins for which the corresponding assigned lowest common ancestor was of higher taxonomic level than class.

**FIGURE 3:**
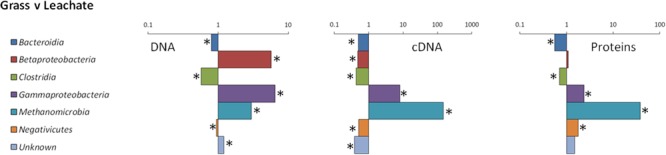
Ratios of phylogenetic assignments in grass biofilm and leachate datasets. Ratios were calculated using (*n*_c_/*n*)/(*N*_c_/*N*), where *n*_c_ is the number of hits (OTUs or proteins) to a given phylogenetic assignment in the grass datasets, *n* is the total number of hits in the corresponding grass datasets (DNA, cDNA, and proteins), *N*_c_ is the number of hits to that phylogenetic assignment in the leachate datasets, and *N* is the total number of hits in the corresponding leachate datasets. Statistically significant over- and under-representation of a given phylogenetic assignment between two datasets is represented by asterisks and was determined by pairwise comparisons using two-tailed Fishers’ exact test with confidence intervals at 99% significance (*P*_adj_ < 0.05).

16S rRNA profiling revealed a total of 1549 operational taxonomic units (OTUs) across the 24 samples analyzed (duplicate grass and leachate DNA, and cDNA, from the triplicate bioreactors). NMDS was used to visualize microbial community dissimilarities between the samples (stress value: 0.095; **Figure 1**). NMDS ordination positions each sample as a function of its distance from all other data points. An NMDS plot stress value below 0.1 indicates that the two-dimensional representation is ideal for data interpretation (Rees et al., 2004). Sample clustering was visually uncovered as a function of (i) bioreactor fraction, i.e., grass or leachate; and (ii) nucleic acid fraction, i.e., DNA or cDNA (**Figure 1**). The observed sample groupings were found to be statistically significant using ANOSIM, with the exception of DNA samples for which grass and leachate bioreactor fractions could not be satisfactorily distinguished (*P*_adj_> 0.05; **Figure 1**). ANOSIM analysis also indicated that the samples did not cluster as a function of bioreactors (*P*_adj_ > 0.05; data not shown). Taken together, the results suggest that molecular (i.e., DNA and cDNA) and bioreactor (i.e., grass and leachate) fractions were the main drivers of microbial community structure. Six taxa, namely Bacteroidia, Betaproteobacteria, Clostridia, Gammaproteobacteria, Methanomicrobia, and Negativicutes, in addition to sequences classified as unknown, accounted for up to 93% of OTUs in the DNA, and 98% in the cDNA, datasets (**Figures 2A,B**). Proteins were also assigned predominantly to these six phylogenetic classes (**Figure 2C**) with only a few proteins assigned to unknown species (**Figure 2C**), as protein-coding sequences from unknown microorganisms are unlikely to be present in the NCBInr database. In addition, the overall contribution of these six phylogenetic classes to protein assignment was less than the contribution to DNA and cDNA. This was attributed to a large proportion of proteins that shared a lowest common ancestor at a taxonomic level higher than class (indicated in brackets in **Figure 2C**). Unclassified OTUs accounted for up to 50% of the microbial community in cDNA samples, highlighting the likely contribution of yet unknown species to the AD of grass (**Figure 2B**). Overall, differences in the relative abundance of the six main taxa could be observed amongst the three molecular datasets (i.e., DNA, cDNA, and proteins). For example, Bacteroidia’s relative abundance was higher in DNA and protein datasets compared to cDNA samples. Conversely, the reverse trend was observed for Negativicutes with an increased relative abundance amongst cDNA and protein datasets compared to DNA samples. Differences could also be seen between grass and leachate fractions at the three levels of molecular investigation (DNA, cDNA, and proteins, **Figures 2, 3**). Bacteroidia and Clostridia were found to be over-represented in the leachate compared to grass biofilms in DNA, cDNA, and protein samples (**Figure 3**). Similarly, Gammaproteobacteria and Methanomicrobia were over-represented in the grass biofilm datasets across the three level of molecular information (**Figure 3**). Negativicutes were under-represented in the grass fraction in both DNA and cDNA datasets while a statistically significant increased number of proteins were assigned to this taxon in the grass fraction when compared to leachate samples (**Figure 3**). This observation is unlikely resulting from a bias in genome availability, as only 84 Negativicutes full genomes are currently available in the NCBI database against, for example, over 1880 Gammaproteobacteria genomes. Finally, Betaproteobacteria and OTUs classified as unknown displayed opposite trends in DNA and cDNA datasets where they were over-represented in the leachate and in the grass fractions, respectively (**Figure 3**). For these two microbial groups no statistically significant differential distribution between grass and leachate fractions could be identified in the protein samples.

### Overview of Microbial Functions

**FIGURE 4:**
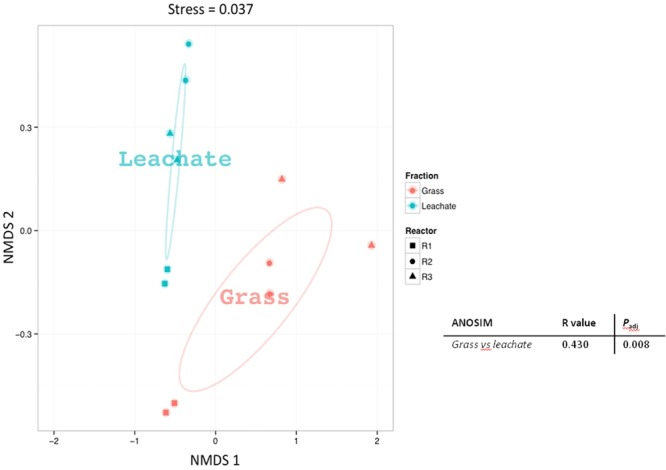
Non-metric multidimensional scaling (NMDS) plot based on Bray–Curtis distances of the proteins extracted from grass biofilm (represented in red) and leachate (represented in blue) fractions from bioreactors R1 (squares), R2 (circles), and R3 (triangles). Analysis of similarity (ANOSIM) was carried out to assess the statistical significance of sample groupings and the corresponding *R*-values and corrected *P*_adj_-values are displayed.

**FIGURE 5:**
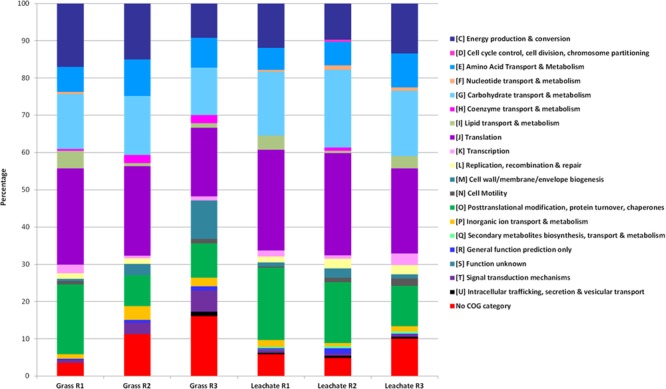
Distribution of functional COG categories in the grass biofilm and leachate metaproteomes. R1, R2, and R3 stands for reactor 1, reactor 2, and reactor 3.

A total of 1,830 proteins was detected across the 12 samples analyzed (duplicate leachate and grass extracts from the triplicate bioreactors; **Supplementary Table S3**), providing an overview of the metabolic pathways likely to be active at the time of sampling (**Supplementary Figure S1**). Grass is typically composed of hemicellulose (ranging from 35 to 50%), cellulose (25 to 40%), lignin (10 to 30%), free sugars (10 to 26%), and lipids (3%; Koch et al., 2010; Ellis et al., 2012). Evidence of the breakdown of grass components was observed in the metaproteomes. Proteins involved in carbohydrate metabolism (including cellulose and hemicellulose degradation, glycolysis and pentose phosphate pathway), energy metabolism (including TCA cycle, methanogenesis and lactate biosynthesis), lipid metabolism (including propionate metabolism, fatty acid β-oxidation and butanol production), amino acid metabolism (including glutamate fermentation to butyrate and nitrogen metabolism), nucleotide metabolism (including purine metabolism), as well as cofactors, and vitamins metabolism (including vitamin B6 biosynthesis) were detected in the triplicate bioreactors (**Supplementary Figure S1** and **Supplementary Table S3**). Sample dissimilarities were assessed with NMDS and ANOSIM, which indicated a clustering of the metaproteomic datasets as a function of bioreactor fraction, i.e., grass biofilms or leachate (stress value: 0.037 and *P*_adj_ < 0.05; **Figure 4**). Classifying microbial proteins into broad functional categories (i.e., cluster of orthologous genes; COG) did not, however, result in any statistically significant differential distribution between the grass and leachate metaproteomes (**Figure 5** and data not shown). The most abundant COG categories, collectively accounting for ∼80% of both grass and leachate proteins, were the following: translation (J), energy production and conversion (C), amino acid transport and metabolism (E), post-translational modification, protein turnover and chaperones (O) and carbohydrate transport and metabolism (G; **Figure 5**).

### Grass Biodegradation

**FIGURE 6:**
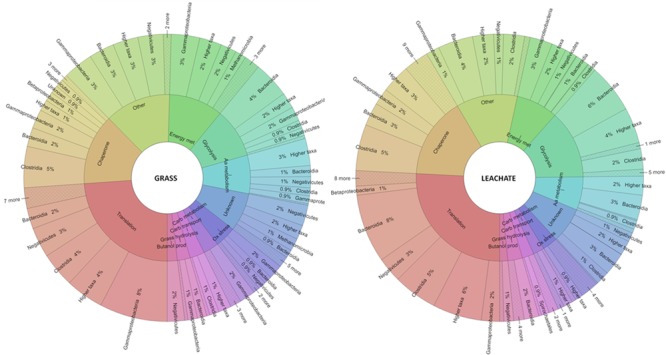
Krona plots displaying phylogenetic classification of proteins detected at the class level on the outer ring and functional key words on the inner ring. aa, amino acid; carb, carbohydrate; met, metabolism; ox, oxidative; prod, production. For an interactive version of the Krona plot see Section “Data Accessibility.”

Evidence of cellulose (i.e., cellobiose hydrolysis) and hemicellulose (i.e., galactose, glycolate, xylan, and xylose catabolism) biodegradation was reflected in both grass and leachate metaproteomes (**Figure 6** and **Supplementary Table S3**). In addition, proteins involved in uronic acid degradation, a component of grass cell wall, could also be detected in both sample types (i.e., grass biofilms and leachate). A cellulose hydrolase (beta-glucosidase) assigned to Bacteroidetes and a xylan hydrolase (beta-1,4-xylanase) assigned to *Clostridium sp*. could only be detected in the leachate samples (**Supplementary Table S3**). It is worth noting, however, that the non-detection of a protein does not necessarily imply that it was not expressed at the time of sampling. A galactose hydrolysing enzyme (beta-galactosidase), exclusively assigned to *E. coli* and a *Megasphaera elsdenii* protein involved in glycolate metabolism were detected in both bioreactor fractions (**Supplementary Table S3**). Xylose degradation was attributed to Bacteroidia in grass biofilms, in addition to Clostridia in the leachate samples (**Figure 6** and **Supplementary Table S3**). Numerous proteins with functions in carbohydrate transport were also detected in all the samples analyzed and mainly assigned to Clostridia and Spirochaetales. Glycolysis was found to take place in both grass and leachate fractions as indicated by a plethora of glycolytic enzymes predominantly assigned to Bacteroidia, Clostridia and Gammaproteobacteria (**Figure 6** and **Supplementary Table S3**).

### Energy Production and Conversion

Many electron transfer proteins were found to be expressed at the time of sampling and were assigned to *Megasphaera* in both bioreactor fractions in addition to *Pseudomonas sp* in the grass biofilms and *Clostridium sp* in the leachate (**Figure 6** and **Supplementary Table S3**). Oxidoreductases were mainly assigned to *Megasphaera elsdenii, Prevotella*, and Clostridiales in all the samples analyzed (**Figure 6** and **Supplementary Table S3**). Numerous ATPases (ABC-type sugar transporter and F-type) and ATP synthases were detected in the triplicate bioreactors and assigned to Gammaproteobacteria, Negativicutes, Bacteroidia and Betaproteobacteria in all samples in addition to Clostridia specifically in the leachate fractions (**Figure 6** and **Supplementary Table S3**). Proteins with functions in the TCA cycle were expressed at the time of sampling and assigned to Gammaproteobacteria in the grass biofilms in addition to Bacteroidia in the leachate samples (**Figure 6** and **Supplementary Table S3**). Enzymes involved in the degradation of propionate to pyruvate and in the conversion of pyruvate to acetyl-coA/acetate were detected in both bioreactor fractions. Acetate could then partly be used as a substrate for methanogenesis as suggested by the detection of enzymes from the corresponding metabolic pathway and assigned to *Methanosarcina* in both leachate samples and grass biofilms (**Figure 6** and **Supplementary Table S3**). Lactaldehyde dehydrogenase, involved in the production of lactate, was assigned to *Dysgonomonas gadei* and detected in the leachate only (**Supplementary Table S3**). Of particular note, proteins involved in the production and conversion of energy that were assigned to Clostridia were only detected in the leachate samples suggesting a possibly more important role for this microbial group in that bioreactor fraction compared to grass biofilms (**Figure 6** and **Supplementary Table S3**).

### Lipid and Amino Acid Metabolism

Fatty acid β oxidation and pyruvate fermentation to butanol were found to take place in both leachate and grass bioreactor fractions (**Figure 6** and **Supplementary Table S3**). Butanol production was exclusively attributed to *Megasphaera elsdenii* in the grass biofilms, in addition to Clostridia in leachate samples. Propionate degradation occurred in the leachate as evidenced by the detection of proteins involved in this metabolic pathway, namely methylmalonyl-CoA mutases assigned to Clostridia and propionyl-CoA carboxylases assigned to Bacteroidia (**Figure 6** and **Supplementary Table S3**). Proteins involved in lipid metabolism that were assigned to Clostridia were only detected in the leachate samples. Proteins assigned to this taxon with roles in amino acid metabolism were detected in both grass and leachate bioreactor fractions, where Clostridia were found to be involved in the fermentation of glutamate to butyrate (**Figure 6** and **Supplementary Table S3**). Bacteroidia and Negativicutes were also implicated in this process with the identification of 2-hydroxyglutaryl-CoA dehydratase and numerous glutamate dehydrogenases (**Supplementary Table S3**). Differences in the taxonomic assignment of proteins involved in amino acid transport and metabolism could be seen between the grass and leachate samples, with for example 17 and 12% of proteins assigned to Negativicutes and Gammaproteobacteria in the grass biofilms compared to 2% and none assigned to these taxa in the leachate fraction (see interactive view of the Krona plots).

### Environmental Stresses

Chaperones were the second largest functional group detected in both bioreactor fractions, accounting for 14 and 16% of the proteins identified in the grass biofilms and leachate samples (**Figure 6** and interactive view of Krona plots). Chaperones are essential for protein folding but also for the re-folding of stress-denatured proteins. Numerous GroEL (60 kDa) and GroES (10 kDa) chaperonins were detected and assigned, amongst other taxa, to Clostridia and Bacteroidia (**Supplementary Table S3**). Recently, the co-expression of GroEL-GroES was found to be imperative for the production of a functional xylose isomerase in *Saccharomyces cerevisiae* (Temer et al., 2017). Xylose isomerases were detected in both bioreactor fractions and assigned exclusively to Clostridia and Bacteroidia (**Supplementary Table S3**). Trigger factor proteins, ClpB, DnaK, and heat shock protein 90 (Hsp90) were also found to be expressed in the bioreactors’ grass biofilms and leachate samples (**Supplementary Table S3**). Trigger factors protect protein nascent chains from aggregation, and play important roles in the stabilization of partially folded proteins (Avellaneda et al., 2017). In addition to performing housekeeping functions, DnaK can either reverse or denature stress-induced protein aggregation (Ghazaei, 2017). Hsp90 and ClpB work in tandem with DnaK to fold or re-fold stress-denatured proteins (Schlieker et al., 2002; Nakamoto et al., 2014). Chaperones were mostly assigned to Clostridia in both grass biofilm and leachate datasets, accounting for over 30% of the proteins from that functional category (see interactive view of the Krona plots). It is worth noting that only 14 and 20% of proteins were assigned to Clostridia in the grass biofilm and leachate metaproteomes. Taken together these results suggest that members of this microbial taxon were experiencing some level of stress within the bioreactors at the time of sampling. Proteins involved in oxidative stress were detected in all the samples analyzed where they were mainly assigned to Clostridia, Negativicutes, Bacteroidia, and Gammaproteobacteria (**Supplementary Table S3**). Desulfoferrodoxin, rubrerythrin, rubredoxin, and superoxide dismutase were detected in both bioreactor fractions in addition to alkyl hydroperoxide reductase assigned to Proteobacteria and glutathione peroxidase from *Megasphaera elsdenii* which were only detected in the leachate samples (**Supplementary Table S3**). Desulfoferrodoxin and superoxide dismutase catalyze the conversion of superoxide radicals to hydrogen peroxide, which is then reduced to water by rubrerythrin and rubredoxin with the latter involved in electron transfer during the oxidation process (Coulter and Kurtz, 2001; Staerck et al., 2017). Alkyl hydroperoxide reductase and glutathione peroxidase can also reduce a variety of hydroperoxides including hydrogen peroxide (Lu and Holmgren, 2014). These results suggest that microorganisms present in the bioreactors’ leachates as well as in grass biofilms were undergoing oxidative stress at the time of sampling. This might not, however, reflect *in situ* conditions but might result from the sampling procedure and downstream analyses.

### Other Functional Activities

Evidence of *Clostridium* sp. sporulation was detected in grass biofilms and leachate samples (**Supplementary Table S3**). Specifically, proteins involved in stage V of sporulation were detected in the two bioreactors’ fractions. This sporulation stage is one of the latest of the process and corresponds to the spore outer coat deposition (Al-Hinai et al., 2015). Sporulation is typically triggered by unfavorable environmental conditions including nutrient depletion, accumulation of butyrate and/or butanol as well as oxidative stress (Dürre, 2014; Al-Hinai et al., 2015). Proteins involved in high affinity phosphate uptake were detected in the leachate metaproteomes and assigned exclusively to Betaproteobacteria and Gammaproteobacteria (**Supplementary Table S3**). This observation might suggest that phosphate is limiting in the leachate bioreactor fraction. Interestingly, an aminoacyl-histidine dipeptidase (PepD) was detected in one of the bioreactor leachate samples (**Supplementary Table S3**) and *pepD* has been shown to be up-regulated during phosphate starvation (Henrich et al., 1992), while its over-expression negatively impacts biofilm formation (Brombacher et al., 2003).

## Discussion

**FIGURE 7:**
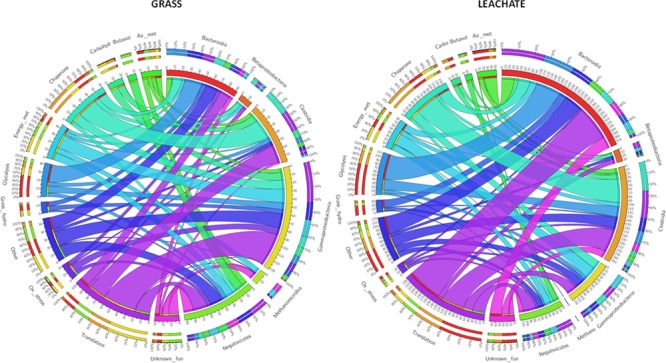
Circos plots displaying broad functional activities and taxonomic assignments on the outer ring and proteins numbers on the inner ring. aa, amino acid; carbohyd, carbohydrate; fun, function; hydro, hydrolysis; met, metabolism; ox, oxidative.

This study investigated microbial community structure and function during the AD of grass, under operating conditions favoring the accumulation of process intermediates. To this end a rigorous experimental strategy encompassing DNA and cDNA 16S rRNA profiling and metaproteomics was deployed on replicated bioreactors. Bioreactor fraction, i.e., grass or leachate, was found to be the main discriminator of community analysis across the three molecular levels of investigation (DNA, RNA, and proteins). Similar microbial groups and functions were detected across the two bioreactor fractions with varying abundance for the 16S rRNA datasets and with changes in phylogenetic assignments for the metaproteomes. Six main taxonomic classes, together with OTUs classified as unknown accounted for the large majority of the three datasets. An overview of the main microbial functions, occurring in the bioreactors at the time of sampling, together with their corresponding phylogenetic assignments is presented in **Figure 7**. Proteins assigned to Gammaproteobacteria and Methanomicrobia represented a larger proportion of the metaproteomes from the grass biofilms when compared to leachate samples, while the reverse was observed for Bacteroidia and Clostridia (**Figure 7**). The same trends were reflected in the DNA and cDNA datasets (**Figure 3**). Taken together these results might indicate a possible preference for biofilm lifestyle as opposed to planktonic for Gammaproteobacteria and Methanomicrobia under the conditions experienced within the bioreactors. Focusing on the anaerobic process of grass acidification, using a similar experimental strategy as the one employed here, whereby acidification resulted from microbial activities, Abendroth et al. (2017) reported a dominance of Bacteroidetes (including Bacteroidia) at 37°C and of Firmicutes (including Clostridia) at 55°C in bioreactor leachates. Similar observations were reported in biogas plants using energy crops as feedstock, where Clostridia and Bacteroidales showed a higher abundance in thermophilic and mesophilic conditions, respectively (Kohrs et al., 2014). Conversely, the phylogenetic class Clostridia was proposed as a marker for biogas plants operated at mesophilic temperatures (Heyer et al., 2016). The prevalence of Clostridiales was also noted during the anaerobic conversion of office paper (mainly composed of cellulose and hemicellulose) to methane at 55°C, during which a rapid decrease from pH 7 to pH 5.8 resulting from hydrolytic and acidogenic microbial activities was reported (Lü et al., 2014). Furthermore, in agreement with the present study, the metaproteomes of the microbial communities involved in cellulose methanisation were dominated by proteins with roles in energy production and conversion (COG category C), carbohydrate transport and metabolism (G) and amino acid transport and metabolism (E; Lü et al., 2014). In addition, evidence of lactate and butanol production was suggested by the detection of enzymes involved in the corresponding metabolic pathways and assigned to *Clostridium* (Lü et al., 2014). Here, butanol production was exclusively attributed to *Megasphaera elsdenii* in the grass biofilms in addition to Clostridiales in the leachate samples (**Figure 7** and **Supplementary Table S1**). Additionally, a lactaldehyde dehydrogenase from *Dysgonomonas gadei* (Bacteroidia), indicative of lactate production, was detected in the leachate bioreactor fractions (**Supplementary Table S3**). Lactate fermentation was also reported in biogas plants with the detection of *E. coli* and Lactobacillales enzymes involved in this metabolic pathway (Kohrs et al., 2014; Heyer et al., 2016). Lactate is also likely degraded and/or involved into the production of medium chain carboxylates within the bioreactors (Zhu et al., 2015) but no evidence of such processes were obtained in the metaproteomes. Butyrate fermentation driven by Bacillales was found to take place during the AD of energy crops, while in the present study this process was mainly attributed to Clostridia, Bacteroidia, and Negativicutes (**Supplementary Table S3**). Clostridia, accounting for 14 and 20% of the grass and leachate metaproteomes, were involved in the initial stages of grass hydrolysis only in the leachate samples, while Bacteroidia, Gammaproteobacteria, and Negativicutes were implicated in this process in the grass biofilms (**Figure 7**). Overall a very similar distribution of functional activities was observed in grass biofilms and leachate samples (**Figures 5, 7**). This, combined with differences in phylogenetic assignment distribution is indicative of functional redundancy whereby the same microbial functions are taking place throughout the replicated bioreactors but are driven by different microbial taxa. It is worth noting that sampling earlier in the bioreactor run might have led to a different conclusion. Evidence of environmental stress conditions prevailing within the bioreactors could be obtained in the metaproteomes. Clostridia seemed particularly affected as suggested by the expression of multiple chaperones, as well as proteins involved in oxidative stress response and sporulation (**Figure 7**). Even though several microbial groups of Methanomicrobia were identified in the DNA and cDNA datasets, including *Methanobacterium, Methanobrevibacter*, and *Methanosaeta*, only proteins from Methanosarcineae were detected in the bioreactors (**Supplementary Table S1**). This observation, together with the detection of stress response proteins, might point to the prevalence of inhospitable environmental conditions at the time of sampling. Indeed, Methanosarcineae have been shown to thrive under sub-optimal anaerobic bioreactor operating conditions (De Vrieze et al., 2012). Overall, this study emphasizes the importance of Clostridia in the AD of grass while highlighting that microbial members from this class were, at least at the time of sampling, experiencing somewhat stressful conditions. Thus, in an effort to optimize the process of grass AD, research avenues aiming at tailoring bioreactor environmental conditions to Clostridia should be explored.

## Data Accessibility

16SrRNA sequence data were deposited on NCBI’s Sequence Read Archive under the accession number SRP119456. Metaproteomic data were deposited to the ProteomeXchange Consortium via the PRIDE partner repository (dataset identifier PXD007956). An interactive view of the Krona plots from **Figure 6** can be accessed here: https://htmlpreview.github.io/?https://github.com/FlorenceAbram/Grass-AD-study/blob/master/Krona_Metaproteomics.html.

## Author Contributions

FA designed the research. AV ran the bioreactors. AJ carried out the biomolecule co-extraction and prepared all samples for downstreams analyses. SS and CB performed the MS analysis. UI, AJ, FA, and CQ carried out the bioinformatic analyses. FA, AJ, and CN analyzed the data. FA and AJ wrote the paper with input from VO.

## Conflict of Interest Statement

The authors declare that the research was conducted in the absence of any commercial or financial relationships that could be construed as a potential conflict of interest.

## Footnotes

1 http://ab.inf.uni-tuebingen.de/software/megan5/

## Supplementary Material

FIGURE S1 Overview of the metabolic pathways occurring at the time of sampling in the triplicate bioreactors (R1, R2, and R3). Red lines indicate reactions catalyzed by enzymes detected in the present study.

TABLE S1 Summary of bioreactor R1, R2, and R3 operational performance.

TABLE S2 Summary of number of MS/MS acquired, MS/MS assigned to peptides and number of distinct peptides for each sample.

TABLE S3 Proteins identified in the 12 samples analyzed. Each technical replicates were combined to give a composite list for each of the triplicate bioreactor fractions: R1, R2, and R3 correspond to bioreactors 1, 2, and 3 and G and L stands for grass biofilm and leachate samples. Name, phylogenetic assignment (class and lowest common ancestor), COG category, predicted function and corresponding accession numbers are displayed for each protein.
