# Supplementary figures and images for: Linking Microbial Community Structure and Function During the Acidified Anaerobic Digestion of Grass
Source: Front Microbiol. 2018 Mar 21;9:540. doi: 10.3389/fmicb.2018.00540 (PMC5871674; doi:10.3389/fmicb.2018.00540)

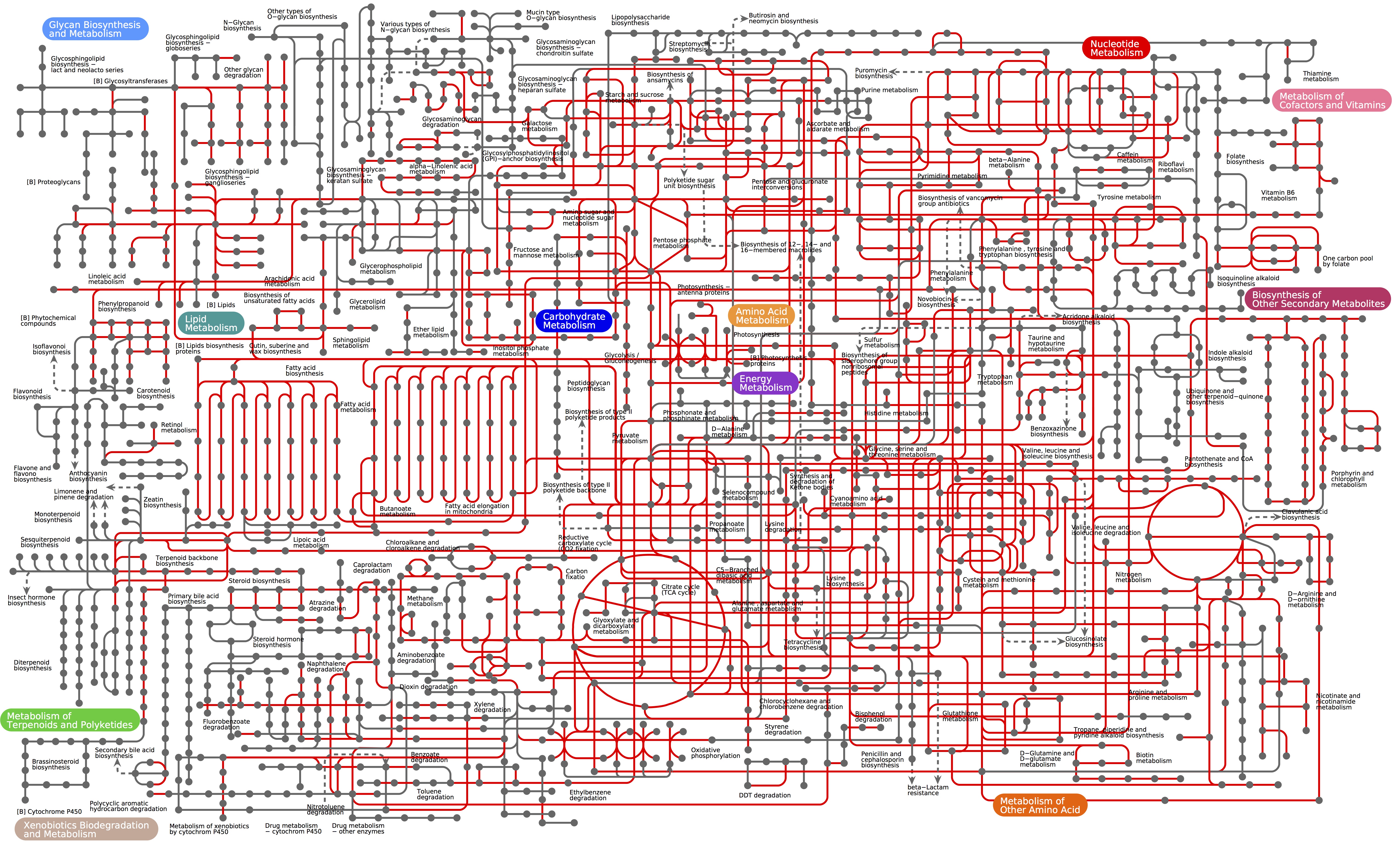

Supplement: FIGURE S1 — Overview of the metabolic pathways occurring at the time of sampling in the triplicate bioreactors (R1, R2, and R3). Red lines indicate reactions catalyzed by enzymes detected in the present study. [file Image_1.JPEG]
