# Supplementary material for: Linking Microbial Community Structure and Function During the Acidified Anaerobic Digestion of Grass
Source: Front Microbiol. 2018 Mar 21;9:540. doi: 10.3389/fmicb.2018.00540 (PMC5871674; doi:10.3389/fmicb.2018.00540)
Supplement: TABLE S1 — Summary of bioreactor R1, R2, and R3 operational performance. [file Table_1.DOCX]

**Table S1**: Summary of bioreactor R1, R2 and R3 operational performance.

| **Bioreactor** | **ml CH_4_ VS g^-1^** | **% CH_4_ in biogas** | **pH** | **sCOD^a^ g L^-1^** |
| --- | --- | --- | --- | --- |
| R1 | 8.31 | 30.7 | 5.5 | 4.4 |
| R2 | 2.59 | 5.9 | 6.3 | 2.2 |
| R3 | 16.1 | 39.7 | 5.7 | 3.4 |

^a^sCOD stands for soluble chemical oxygen demand
