# Supplementary material for: Linking Microbial Community Structure and Function During the Acidified Anaerobic Digestion of Grass
Source: Front Microbiol. 2018 Mar 21;9:540. doi: 10.3389/fmicb.2018.00540 (PMC5871674; doi:10.3389/fmicb.2018.00540)
Supplement: TABLE S2 — Summary of number of MS/MS acquired, MS/MS assigned to peptides and number of distinct peptides for each sample. [file Table_2.DOC]

**Table S2**: Summary of number of MS/MS acquired, MS/MS assigned to peptides and number of distinct peptides for each sample.

| Reactor | Sample type | Replicate | Gel chunk No.:  Region of gel track | No. MS/MS acquired | No. MS/MS assigned  to peptides (>0.05) | No. different peptide  sequences (>0.05) |
| --- | --- | --- | --- | --- | --- | --- |
| 1 | grass | A | 1: upper | 5289 | 1377 | 1148 |
| 1 | grass | A | 2: middle | 5639 | 1746 | 1421 |
| 1 | grass | A | 3: lower | 5508 | 1676 | 1413 |
| 1 | grass | B | 4: upper | 9585 | 2625 | 2049 |
| 1 | grass | B | 5: middle | 7615 | 2716 | 2052 |
| 1 | grass | B | 6: lower | 8211 | 3169 | 2462 |
| 1 | leachate | A | 7: upper | 10722 | 2734 | 2077 |
| 1 | leachate | A | 8: middle | 8785 | 2876 | 2058 |
| 1 | leachate | A | 9: lower | 9628 | 3229 | 2492 |
| 1 | leachate | B | 10: upper | 10757 | 2453 | 1828 |
| 1 | leachate | B | 11: middle | 9109 | 2797 | 1985 |
| 1 | leachate | B | 12: lower | 10580 | 3312 | 2492 |
| 2 | grass | A | 13: upper | 8269 | 1699 | 1157 |
| 2 | grass | A | 14: middle | 5909 | 1367 | 958 |
| 2 | grass | A | 15: lower | 8871 | 2265 | 1728 |
| 2 | grass | B | 16: upper | 9336 | 1856 | 1124 |
| 2 | grass | B | 17: middle | 7531 | 1258 | 755 |
| 2 | grass | B | 18: lower | 10069 | 2423 | 1785 |
| 2 | leachate | A | 19: upper | 10813 | 2308 | 1633 |
| 2 | leachate | A | 20: middle | 9360 | 2309 | 1589 |
| 2 | leachate | A | 21: lower | 12469 | 2549 | 1809 |
| 2 | leachate | B | 22: upper | 11624 | 2336 | 1644 |
| 2 | leachate | B | 23: middle | 11109 | 3004 | 2065 |
| 2 | leachate | B | 24: lower | 13172 | 3204 | 2298 |
| 3 | grass | A | 25: upper | 7560 | 2299 | 1333 |
| 3 | grass | A | 26: middle | 5859 | 1403 | 865 |
| 3 | grass | A | 27: lower | 8157 | 1982 | 1382 |
| 3 | grass | B | 28: upper | 4388 | 952 | 507 |
| 3 | grass | B | 29: middle | 5447 | 1047 | 562 |
| 3 | grass | B | 30: lower | 6031 | 1257 | 785 |
| 3 | leachate | A | 31: upper | 11323 | 2304 | 1628 |
| 3 | leachate | A | 32: middle | 9063 | 2560 | 1764 |
| 3 | leachate | A | 33: lower | 10433 | 2640 | 1929 |
| 3 | leachate | B | 34: upper | 10139 | 2108 | 1491 |
| 3 | leachate | B | 35: middle | 8118 | 2120 | 1487 |
| 3 | leachate | B | 36: lower | 11421 | 2860 | 2004 |
